# Excessive occupational sitting increases risk of cardiovascular events among working individuals with type 1 diabetes in the prospective Finnish Diabetic Nephropathy Study

**DOI:** 10.1186/s12933-024-02486-7

**Published:** 2024-10-29

**Authors:** Matias Seppälä, Heidi Lukander, Johan Wadén, Marika I. Eriksson, Valma Harjutsalo, Per-Henrik Groop, Lena M. Thorn, FinnDiane Study Group

**Affiliations:** 1 Folkhälsan Research Center, Biomedicum Helsinki, Helsinki, Finland; 2 Research Program for Clinical and Molecular Metabolism, University of Helsinki, Helsinki, Finland; 3 Department of Nephrology, University of Helsinki and Helsinki University Hospital, Helsinki, Finland; 4 Department of Diabetes, Central Clinical School, Monash University, Melbourne, VIC Australia; 5 Department of General Practice and Primary Health Care, University of Helsinki and Helsinki University Hospital, PoB 20, 00014 Helsinki, Finland

**Keywords:** Occupational sitting, Sedentary behavior, Cardiovascular events, All-cause mortality, Type 1 diabetes

## Abstract

**Background:**

Sedentary behavior, such as excessive sitting, increases risk of cardiovascular disease and premature mortality in the general population, but this has not been assessed in type 1 diabetes. Occupational sitting is increasingly ubiquitous and often constitutes the largest portion of daily sitting time. Our aim was to identify clinical factors associated with excessive occupational sitting in type 1 diabetes and, in a prospective setting, to explore its association with cardiovascular events and all-cause mortality, independent of leisure-time physical activity.

**Methods:**

An observational follow-up study of 1,704 individuals (mean age 38.9 ± 10.1 years) from the Finnish Diabetic Nephropathy Study. Excessive occupational sitting, defined as ≥ 6 h of daily workplace sitting, was assessed using a validated self-report questionnaire. Data on cardiovascular events and mortality were retrieved from national registries. Multivariable logistic regression identified independently associated factors, while Kaplan-Meier curves and Cox proportional hazard models were used for prospective analyses.

**Results:**

Factors independently and positively associated with excessive occupational sitting included a high occupational category [OR 6.53, 95% CI (4.09‒10.40)] and older age [1.02 (1.00‒1.03)], whereas negatively associated factors included current smoking [0.68 (0.50‒0.92)], moderate albuminuria [0.55 (0.38‒0.80)], and high leisure-time physical activity [0.52 (0.36‒0.74)]. During a median follow-up of 12.5 (6.5–16.4) years, 163 individuals (9.6%) suffered cardiovascular events, and during a median follow-up of 13.7 (9.4–16.6) years, 108 (6.3%) deaths occurred. Excessive occupational sitting increased cardiovascular event risk (hazard ratio [HR] 1.55 [95% CI 1.10‒2.18]) after adjustment for confounders and other covariates. Furthermore, in a stratified multivariable analysis among current smokers, excessive occupational sitting increased the risk of all-cause mortality (2.06 [1.02‒4.20]).

**Conclusions:**

Excessive occupational sitting is associated with a higher risk of cardiovascular events and all-cause mortality in individuals with type 1 diabetes. This association persists regardless of leisure-time physical activity, after adjusting for independently associated variables identified in our cross-sectional analyses. These findings underscore the need to update physical activity guidelines to better address sedentary behavior and improve outcomes for individuals with type 1 diabetes. Targeting occupational sitting should be considered a key focus for interventions aimed at reducing overall sedentary time.

**Graphical Abstract:**

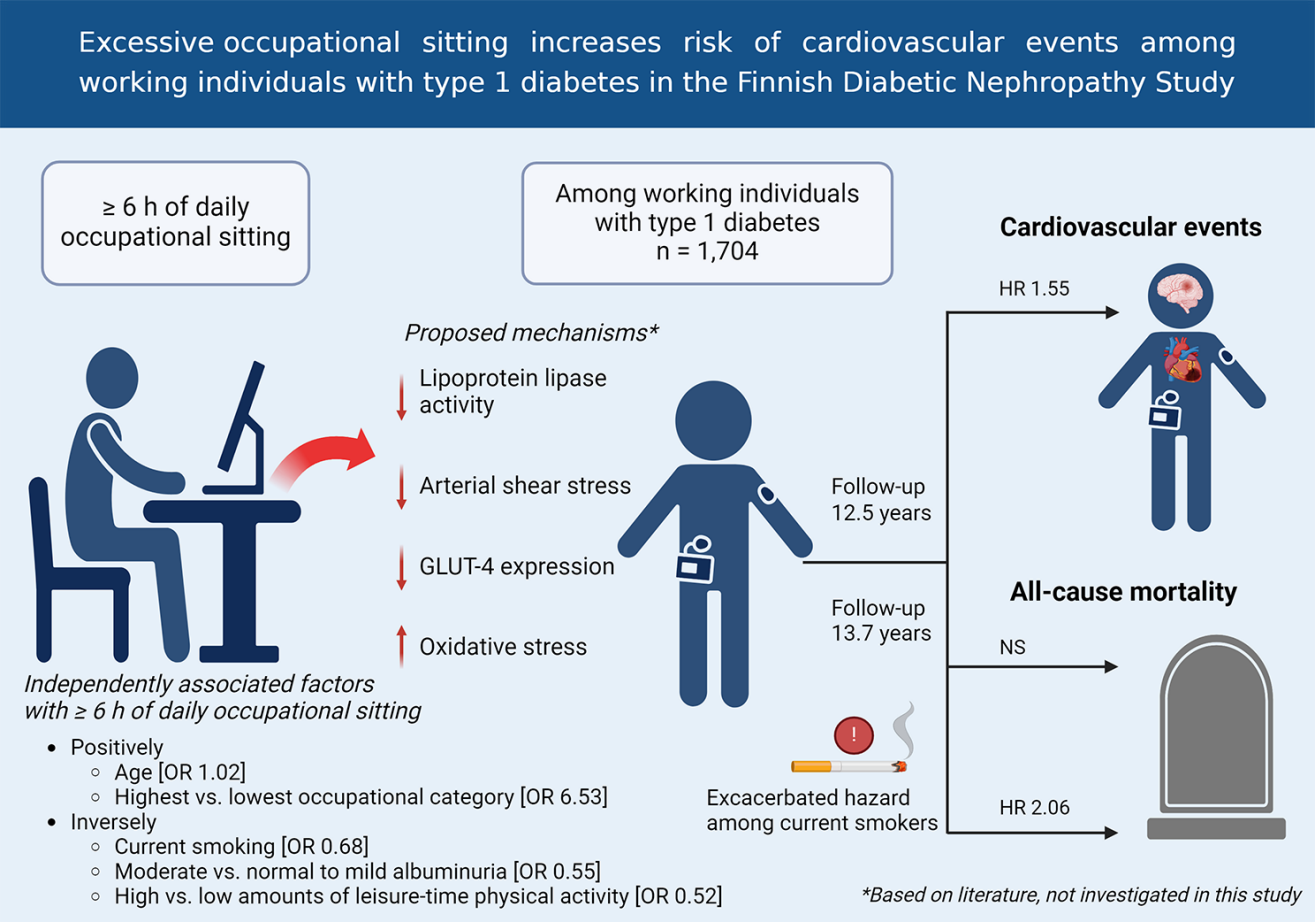

**Supplementary Information:**

The online version contains supplementary material available at 10.1186/s12933-024-02486-7.

## Background

Physical activity offers significant health benefits for individuals with type 1 diabetes (T1D), including reductions in micro- and macrovascular complications and lower premature mortality [1–4]. However, being physically active does not preclude accumulating sedentary behavior, defined as any waking behavior with a low energy expenditure (≤ 1.5 metabolic equivalents) while in sitting, reclining, or lying posture [5]. In the general population, excessive sitting and other forms of sedentary behavior are associated with chronic non-communicable diseases such as cardiovascular disease and premature mortality, largely independent of physical activity [6, 7]. This has translated into a paradigm shift in global physical activity guidelines, emphasizing not only the importance of achieving sufficient moderate-to-vigorous physical activity (MVPA) but also limiting sedentary behavior [8].

In the context of diabetes, the American Diabetes Association’s position statement on physical activity recommends that all adults, particularly those with type 2 diabetes (T2D), reduce daily sedentary behavior. It further advises interrupting prolonged periods of sitting with light physical activity every 30 min, at least for adults with T2D [9]. However, these guidelines primarily address T2D, and do not explicitly communicate the need for reducing sedentary behavior in individuals with T1D.

Notably, individuals with T1D also experience high levels of sedentary behavior, often more so than those without diabetes [10]. Furthermore, most individuals with T1D do not meet the physical activity guidelines [11], which is concerning, as evidence from the general population suggests that sedentary behavior is particularly harmful to those who engage in low levels of MVPA [12]. Another high-risk group among individuals with T1D where sedentary behavior may be especially harmful is current smokers. A recent meta-analysis demonstrated that smoking could modify the relationship between sedentary behavior and all-cause mortality in the general population [13]. This is relevant given that the prevalence of smoking among those with T1D is significant, only slightly lower compared to the general population [14].

Given the high prevalence of sedentary behavior and physical inactivity among individuals with T1D, there is an urgent need to develop clear and targeted guidelines for managing sedentary behavior in this population. The current guidelines are based on studies involving the general population or individuals with T2D or the risk of developing T2D, with limited research focusing specifically on T1D. Cross-sectional findings indicate an association between sedentary behavior and higher glycated hemoglobin (HbA_1c_) levels among children and adolescents with T1D [15]. Recently, an interventional study demonstrated that interrupting prolonged sitting with bouts of light-intensity activity improved postprandial glucose levels and time in range, with sustained improvement up to 48 h [16]. However, there are no prospective studies on the impact of sedentary behavior and long-term health outcomes among individuals with T1D.

Among various domains of sedentary behavior, occupational sedentary behavior (i.e., occupational sitting) has become increasingly relevant as job tasks have become more sedentary in recent decades [17, 18]. Occupational sitting may be particularly hazardous not only due to its growing prevalence but also because of factors beyond personal control, such as organizational culture, workplace environment, and competing priorities. These factors can increase total sitting time and promote prolonged sedentary behavior [19, 20]. Nevertheless, the workplace also presents a promising setting for reducing sedentary behavior, as interventions have proven notably effective in decreasing occupational sitting compared to other sedentary behavior domains [21]. If research confirms the hazardous effects of occupational sitting or sedentary behavior in general among individuals with T1D, limiting sedentary time in the workplace could be a pragmatic strategy to mitigate these risks in this population.

To our knowledge, there is no previous literature on the association between occupational sitting time, or sedentary behavior in general, and long-term health hazards and mortality in T1D. Therefore, the aims of this study were (1) in a cross-sectional setting to identify which clinical, diabetes-related, and sociodemographic factors are associated with excessive occupational sitting in T1D, and (2) in a prospective setting to examine the association between excessive occupational sitting and cardiovascular events and all-cause mortality in individuals with T1D, independently of physical activity.

## Methods

### Participants

All individuals are participants in the nationwide, multicenter, Finnish Diabetic Nephropathy (FinnDiane) Study, founded in 1997. The aim of the study is to uncover genetic, environmental, and clinical risk factors for micro- and macrovascular complications of T1D. The research design and study protocol have previously been described in detail [22]. The recruitment of participants and data collection occurred at regular visits to the attending physician in 77 study centers across Finland (Supplementary Table 1). In this substudy, we identified 2,725 individuals in the FinnDiane database with T1D and a questionnaire regarding data on occupational sitting and leisure-time physical activity available. T1D was defined as diabetes onset before 40 years of age, and initiation of insulin therapy within one year from diagnosis. Out of these individuals, 831 were excluded due to missing occupational data, mainly because they were receiving disability pension, were unemployed, or retired, and thus not actively part of the workforce at the baseline visit. No individuals were excluded based on their occupation. Moreover, 129 individuals were excluded either due to missing information on their workday length or because they worked less than 6 h per day. This cutoff was used as a proxy to differentiate between full-time and part-time employment, due to the lack of direct data on work status. It corresponds to less than 30 h per week based on a typical 5-day workweek, which is commonly considered the threshold for full-time employment in Finland [23]. Finally, 61 individuals were excluded due to missing data on kidney status, leaving us with 1,704 eligible individuals for the study. The individuals in the excluded population tended to be older, had a longer duration of diabetes, higher systolic blood pressure, higher triglycerides, more prevalent use of lipid-lowering medication, belonged more frequently to the lowest occupational category, and had a higher prevalence of diabetic complications including diabetic kidney disease and retinal photocoagulation. On the other hand, individuals in the excluded population accumulated more frequently high amounts of leisure-time physical activity and were less frequently current smokers (Supplementary Table 2).

### Ethical considerations

The study protocol was approved by the Ethics Committee of the Helsinki and Uusimaa Hospital District, and the study was conducted in accordance with the Declaration of Helsinki. Each participant provided their written informed consent.

### Baseline study visit

Information regarding medical history, current medication, cardiovascular status, and diabetic complications were registered. Anthropometric data (body weight, height, waist-, and hip circumferences) and office blood pressure measurements were recorded at the study visit. In addition, blood samples were drawn and analyzed for HbA_1c_, lipids and lipoproteins, and serum creatinine. Participants collected timed urine samples for the assessment of urinary albumin excretion rate. The kidney status was determined by measurements from two out of three consecutive overnight or 24-hour urine collections. Normal to mild albuminuria was considered < 20 µg/min or < 30 mg/24 h (*n* = 1,289), moderately increased albuminuria 20–200 µg/min or 30–300 mg/24 h (*n* = 218), and severely increased albuminuria > 200 µg/min or > 300 mg/24 h (*n* = 150). The estimated glomerular filtration rate (eGFR) was estimated by the Chronic Kidney Disease Epidemiology Collaboration equation (CKD-EPI) [24]. Diabetic kidney disease (DKD) was defined as a history of severe albuminuria, or kidney replacement therapy. A cardiovascular event was defined as a history of myocardial infarction (*n* = 25), coronary revascularization (*n* = 38), stroke (*n* = 15), amputation (*n* = 13), or peripheral artery revascularization (*n* = 13). Severe diabetic retinopathy (SDR) was defined as a history of retinal photocoagulation. Additionally, the participants filled out questionnaires regarding their medical history, lifestyle habits, and socioeconomic status. Current smoking was defined as smoking at least one cigarette a day for one year prior to data collection. Occupational categories were defined as follows: unskilled blue-collar, skilled blue-collar, lower white-collar, and upper white-collar. In this classification, unskilled blue-collar represents the lowest occupational category. The occupational category variable reflects both participants’ educational level and occupation.

### Assessment of occupational sitting and leisure-time physical activity

Occupational sitting and leisure-time physical activity (LTPA) were assessed from a self-report questionnaire. The validation and details of the questionnaire have been previously described [25, 26]. The questionnaire consists of three sections. The first section evaluates present or earlier LTPA on a general level regarding the type of physical activity, frequency, duration, and intensity of LTPA. The second section assesses more specific information on frequency (times per month), duration per session (minutes), and intensity (on a scale from 0 to 3) for the 21 of the most common Finnish forms of leisure-time physical activities, retrospectively for the past 12 months. Based on the data in the second section, we calculated the total amount of LTPA in metabolic equivalents (MET-h/week), and the participants were categorized as achieving low (< 10 MET-h/week), moderate (10–40 MET-h/week), or high amounts (> 40 MET-h/week), where achieving low amounts of LTPA reflects a failure to meet the general physical activity guidelines [27]. The cutoffs for categories are consistent with those used in our previous studies [4, 28]. The third section of the questionnaire assesses various work-related factors. It includes current employment status, total workday length, and the duration of breaks (e.g., lunch, coffee). This section also collects data on occupational sitting and physical activity, including standing, walking, and climbing stairs. The duration of occupational sitting was estimated by asking participants to report their total sitting time during a typical working day in minutes. Excessive occupational sitting was defined as sitting for ≥ 6 h per day, compared to < 6 h. This cutoff was chosen based on data patterns and theoretical considerations. Sitting time was categorized into tertiles, rounded to the nearest whole hour, with comparisons made between the highest tertile and the two lower tertiles. The 6-hour threshold corresponds with research indicating that about one-third of the Finnish workforce sits for 6–7 h daily, making it a relevant benchmark [29]. Furthermore, extrapolation from meta-analyses [7, 30] suggest that the critical threshold for the impact of sedentary behavior on cardiovascular events may fall within the 6–7-hour range [31].

### Follow-up data

Data on mortality were obtained from the Finnish Cause of Death Register, Statistics Finland. Data on cardiovascular events (coronary artery disease events, *n* = 121, including acute myocardial infarctions or coronary revascularizations, or stroke, *n* = 42) were obtained from the Finnish Care Register for Health Care, and the Finnish Institute for Health and Welfare based on ICD-10 (codes I21-I23, and I60-I64), ICD-9 (codes 410, 412, and I430-I434), and Nordic Medico-Statistical Committee (NOMESCO) Classification of Surgical Procedures codes FNA, FNB, FNC, FND, FNE, FNF, FNG, TNF40, FN1AT, FN1BT, or FN1YT for procedures before 2016 and FN2 codes for procedures after 2016. Follow-up data were available until the end of 2017 for all outcomes. Follow-up time was calculated from the baseline visit until cardiovascular event, death, or the end of 2017.

### Statistical analyses

All variables were tested for normal distribution by visual inspection. Between-group differences in categorical variables were analyzed with the χ^2^-test. Normally distributed continuous variables were analyzed with Student´s t-test. Non-parametric continuous variables were analyzed with Mann-Whitney U-test. Data are presented as percentages (categorical variables), mean ± standard deviation (normally distributed variables), or median with interquartile range (non-parametric continuous variables). A multiple logistic regression analysis was performed to identify variables independently associated with excessive occupational sitting. Variables were selected for the logistic regression analysis based on theoretical justification and significance (*p* < 0.05) in univariable analyses. The results are presented as odds ratio (OR) with 95% confidence interval (CI). In a prospective setting, we explored the survival or event-free time of excessive occupational sitting (≥ 6 h versus < 6 h) using the Kaplan-Meier method and compared statistically using the log-rank test. Cox proportional hazards analysis was conducted to investigate the independent association between excessive occupational sitting and cardiovascular events and all-cause mortality. The rationale for selecting variables into the multivariable analyses was to include (1) confounders, in other words variables associated with both the exposure (occupational sitting) and the outcomes (cardiovascular events and all-cause mortality), but not resulting from the exposure, and (2) additional covariates that, while not confounders, were considered essential due to their impact on our outcomes. In total, four different models were constructed. Model 1 is an unadjusted model. In Model 2, we adjusted for confounders namely age, occupational category, current smoking, and kidney status. In Model 3, we added essential covariates namely sex, severe retinopathy, earlier cardiovascular event, HbA_1c_, systolic blood pressure, and LDL cholesterol. Finally, in Model 4, we adjusted for moderate-high leisure-time physical activity, reflecting the fulfillment of physical activity guidelines. Results from the Cox regression analyses are presented as hazard ratios (HR) with 95% CI. The proportional hazards assumption was tested using Schoenfeld residuals over time. Current smoking was the only variable that violated this assumption. To address this, we assessed its time-dependent effect and refitted models 2, 3, and 4 to include an interaction term between smoking and time. This adjustment was necessary for cardiovascular events but not for all-cause mortality. Multicollinearity in the models was evaluated by performing linear regression with variance inflation factors (VIF), all values were below three, indicating low risk of multicollinearity.

In our multivariable analyses among current smokers, we selected variables using the same rationale. However, due to the limited number of events, we only adjusted for moderate-high physical activity and key confounders, without including additional covariates. The key confounders included duration of diabetes, occupational category, and diabetic kidney disease. All statistical analyses were performed with SPSS Statistics 27.0 software (IBM Corporation, Armonk, NY, USA).

## Results

The study population included 1,704 individuals with T1D (1,289 with normal to mild albuminuria, 218 with moderate albuminuria, 150 with severely increased albuminuria, and 47 with kidney replacement therapy). Out of these individuals, 48.8% were men, mean age was 38.9 ± 10.1 years, duration of diabetes 22.3 ± 11.7 years, BMI 25.5 ± 3.7 kg/m^2^, systolic blood pressure 133 ± 17 mmHg, HbA_1c_ 8.1 ± 1.3% (65.4 ± 14.3 mmol/mol), and median occupational sitting time 4.0 (1.5–6.0) hours.

The associations between baseline clinical characteristics and occupational sitting are presented in Table 1. Participants with excessive sitting of ≥ 6 h were significantly older, had a longer duration of diabetes, and were less frequently highly physically active during their leisuretime. On the other hand, they were less frequently current smokers, had better glycaemic control, belonged more frequently to the highest occupational category, had more frequently normal to mild albuminuria, and performed more frequently moderate amounts of leisure-time physical activity. There was no association between occupational sitting and sex, BMI, systolic blood pressure, or blood lipid concentrations.

**Table 1:** Clinical characteristics of the study cohort, grouped by occupational sitting

Characteristic	< 6 h occupational sitting *n* = 1,122	Excessive occupational sitting *n* = 582	*p*-value
Men, % (n)	48.4 (543)	46.9 (273)	0.560
Age, years	38.4 ± 10.3	39.8 ± 9.6	0.007
Duration of diabetes, years	21.7 ± 11.5	23.4 ± 12.0	0.007
BMI, kg/m^2^	25.5 ± 3.7	25.6 ± 3.7	0.569
Systolic blood pressure, mmHg	133 ± 16	133 ± 17	0.935
Diastolic blood pressure, mmHg	79 ± 9	79 ± 9	0.337
Total cholesterol, mmol/l	4.79 ± 0.85	4.73 ± 0.89	0.191
LDL cholesterol, mmol/l	2.80 ± 0.78	2.77 ± 0.80	0.483
HDL cholesterol, mmol/l	1.49 ± 0.42	1.47 ± 0.40	0.323
Triglycerides, mmol/l	0.96 (0.74–1.32)	0.91 (0.70–1.30)	0.120
HbA_1c_, %	8.2 ± 1.3	8.0 ± 1.3	0.009
HbA_1c_, mmol/mol	66.1 ± 14.4	64.2 ± 13.9	0.009
Retinal photocoagulation, % (n)	25.7 (286)	22.6 (131)	0.164
Diabetic kidney disease, % (n)	11.8 (132)	11.2 (65)	0.715
Cardiovascular event, % (n)	3.7 (41)	5.3 (31)	0.108
Current smoking, % (n)	26.1 (285)	15.0 (85)	< 0.001
Antihypertensive medication, % (n)	33.6 (375)	31.7 (184)	0.422
Lipid lowering medication, % (n)	16.4 (183)	18.1 (105)	0.388
eGFR, ml/min/1.73 m^2^	106 (91–117)	104 (90–114)	0.019
Kidney status			
Normal to mildly increased albuminuria, % (n)	73.2 (821)	80.4 (468)	< 0.001
Moderately increased albuminuria, % (n)	15.1 (169)	8.4 (49)	< 0.001
Severely increased albuminuria, % (n)	9.1 (102)	8.2 (48)	0.560
Kidney failure with replacement therapy, % (n)	2.7 (30)	2.9 (17)	0.768
Occupational category			
Upper white-collar, % (n)	9.3 (104)	25.4 (148)	< 0.001
Lower white-collar, % (n)	23.4 (263)	34.0 (198)	< 0.001
Skilled blue-collar, % (n)	42.5 (477)	23.7 (138)	< 0.001
Unskilled blue-collar, % (n)	14.3 (160)	6.2 (36)	< 0.001
LTPA			
High amounts of LTPA, % (n)	19.8 (222)	13.9 (81)	0.003
Moderate amounts of LTPA, % (n)	50.6 (568)	57.9 (337)	0.004
Low amounts of LTPA, % (n)	29.6 (332)	28.2 (164)	0.543
Total LTPA, MET-h/week	19.3 (8.1–34.1)	17.6 (9.1–30.3)	0.270

Data are mean ± standard deviation, median (interquartile range), or percentage (%) and n-value (n). eGFR = estimated glomerular filtration, LTPA = Leisure-time physical activity, High amounts of LTPA = > 40 MET-h/week, Moderate amounts of LTPA = 10–40 MET-h/week, Low amounts of LTPA = < 10 MET-h/week. MET-h/week = Metabolic equivalent hours/week.

Further, in a multiple logistic regression analysis exploring independently associated variables, we found that participants with excessive occupational sitting time were older [OR 1.02, (95% CI 1.00–1.03), *p* = 0.009], and more likely to belong to the highest occupational category [OR 6.53, (4.09–10.40), *p* < 0.001] compared with the lowest occupational category. They were less likely to have moderate albuminuria [OR 0.55, (0.38–0.80), *p* = 0.002] compared with normal to mild albuminuria. They were less frequently current smokers [OR 0.68, (0.50–0.92), *p* = 0.012], but did less frequently achieve high amounts of leisure-time physical activity [OR 0.52, (0.36–0.74), *p* < 0.001] compared with low amounts of leisure-time physical activity. The multivariable logistic regression model is presented in Supplementary Table 3.

In the prospective setting of our study, 163 (9.6%) participants developed a cardiovascular event and 108 (6.3%) died of any cause during a median follow-up time of 12.5 (6.5–16.4) and 13.7 (9.4–16.6) years, respectively. Supplementary Table 4 shows the baseline characteristics of the individuals, according to the presence or absence of a given event during follow-up. Individuals who died during follow-up from any cause were older at baseline, more often men, had a longer duration of diabetes, higher blood pressure, unfavorable lipid profile, higher HbA_1c_, lower eGFR, belonged more often to the lowest occupational category, were more often current smokers, were more often physically inactive (low amounts of LTPA), had more frequently a history of diabetic kidney disease, retinal photocoagulation, and a history of cardiovascular events. Individuals who suffered a cardiovascular event shared largely the same characteristics as those who died from any cause. While similar trends regarding sex, current smoking, and lower white-collar were observed, they lacked statistical significance. Moreover, individuals who experienced a cardiovascular event were not more frequently physically inactive. HDL-cholesterol, however, was significantly lower among individuals who suffered a cardiovascular event during follow-up, which was distinct from individuals who died from any cause.

**Fig. 1:**
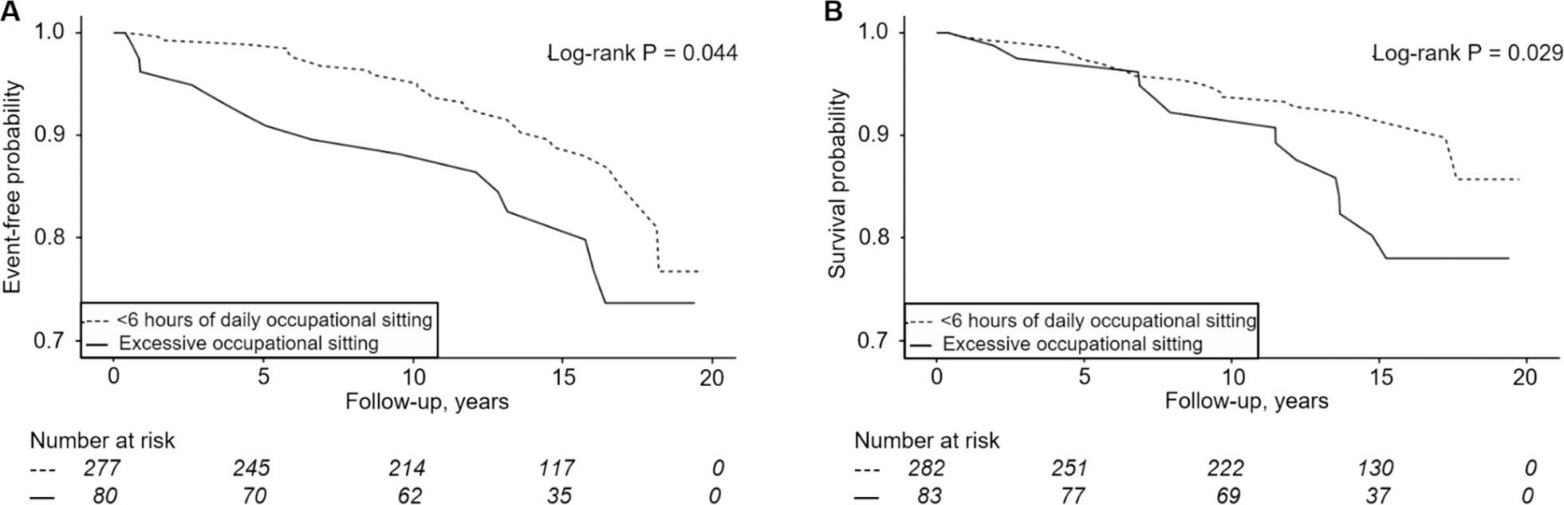
Kaplan-Meier (**A**) cardiovascular event-free and (**B**) survival probabilities for excessive occupational sitting among current smokers

We investigated the impact of excessive occupational sitting on cardiovascular event-free time and survival using the Kaplan-Meier method. In our unstratified models, excessive occupational sitting did not influence our outcomes (Supplementary Fig. 1). In the stratified models among current smokers, however, excessive occupational sitting was associated with decreased cardiovascular event-free time and survival (Fig. 1)*.*

The multivariable Cox proportional hazard regression models exploring the independent association between excessive occupational sitting and cardiovascular events and all-cause mortality are shown in Table 2. Model 1 represents the unadjusted model, where no significant associations were observed. In Model 2, excessive occupational sitting was associated with an increased risk of cardiovascular events, after adjustment for confounders. In Model 3, after further adjustment for covariates, excessive occupational sitting was still associated with an increased risk of cardiovascular events. The association remained significant in the final model (Model 4), where we adjusted for guideline-recommended amounts of leisure-time physical activity. Excessive occupational sitting did not increase the risk of all-cause mortality in any of our four models. However, in a multivariable Cox regression analysis among current smokers, occupational sitting was associated with an increased risk and all-cause mortality after adjustments for duration of diabetes, occupational category, diabetic kidney disease and moderate-high leisure-time physical activity. Although a positive trend remained, the association between excessive occupational sitting and cardiovascular events among current smokers did not remain statistically significant in the final model (Supplementary Table 5).

**Table 2:** Cox regression models showing the association between excessive occupational sitting and cardiovascular events and mortality

Model	Cardiovascular event			All-cause mortality		
	*n* events (*n* in analysis)	HR (95% CI)	*p*-value	*n* events (*n* in analysis)	HR (95% CI)	*p*-value
1	163 (1,614)	1.28 (0.93–1.75)	0.128	108 (1,654)	1.16 (0.79–1.72)	0.450
2	156 (1,570)	1.45(1.04–2.03)	0.031	104 (1,610)	1.28(0.84–1.94)	0.246
3	154 (1,511)	1.53 (1.09–2.16)	0.015	100 (1,522)	1.39 (0.90–2.13)	0.135
4	154 (1,511)	1.55 (1.10–2.18)	0.013	100 (1,522)	1.35 (0.88–2.08)	0.164

Data are hazard ratios (HR) with 95% confidence intervals (CI).

*Model 1*: Unadjusted model. *Model 2*: Model 1 + age, occupational category (upper and lower white-collar *versus* blue-collar), current smoking, interaction term between smoking and time (only for cardiovascular events), kidney status (normal *versus* moderately increased *versus* severely increased albuminuria or kidney replacement therapy). *Model 3*: Model 2 + sex, retinal photocoagulation, previous cardiovascular event, HbA_1c_, systolic blood pressure, and LDL cholesterol. *Model4*: Model 3 + moderate-high LTPA (adjustment for achieving physical activity recommendations).

## Discussion

In this large observational follow-up study of individuals with T1D, our main finding was that excessive occupational sitting is associated with an increased risk of cardiovascular events, independent of leisure-time physical activity. Notably, no such association was observed for all-cause mortality. However, in a subanalysis of current smokers, excessive occupational sitting was linked to an increased risk of all-cause mortality. Additionally, our cross-sectional analysis identified variables independently associated with excessive occupational sitting. Age and higher occupational category were positively associated, while current smoking, moderate albuminuria, and high levels of leisure-time physical activity were negatively associated. Overall, our findings indicate that current physical activity guidelines need updating to better address sedentary behavior and improve outcomes for individuals with T1D. Occupational sitting, as a specific domain of sedentary behavior, should be considered a key area for interventions aimed at reducing overall sedentary time.

This is the first study, to our knowledge, assessing long-term health outcomes of occupational sitting or sedentary behavior in general in T1D.

Our findings in individuals with T1D align with a positive association between sedentary behavior and cardiovascular events in the general population [32]. Despite a significant increase in research over the past decade, evidence regarding occupational sitting as a specific domain of sedentary behavior remains inconclusive [33, 34]. Several factors may contribute to this uncertainty. One key issue is the potential differences between the correlates of occupational sitting and those of leisure-time or total sitting time, which underpin existing conclusive evidence linking sedentary behavior to adverse health outcomes. For instance, while socioeconomic status is positively associated with occupational sitting, the association is inverse for leisure-time sitting [35]. Similarly, current smoking, a fundamental cardiovascular risk factor, is negatively associated with occupational sitting in contrast to leisure-time sedentary and total sedentary time [35, 36]. Consequently, when considering the impact of occupational sitting on adverse health outcomes, socioeconomic status and its related multitude of health factors might offset a positive association without thorough adjustments in the analyses [37]. Our findings among individuals with T1D highlight this complexity. We observed that those with excessive occupational sitting were more likely to be in higher occupational categories—a proxy for socioeconomic status—and less likely to be current smokers, both of which act as protective factors despite the risks associated with excessive occupational sitting. However, not all beneficial lifestyle habits were positively associated with excessive occupational sitting, as these individuals were less frequently highly physically active during leisure time. Another reason for the inconclusive evidence on occupational sitting is the substantial heterogeneity in measurement methods, study designs, and outcome variables. This variability makes it difficult to draw definitive conclusions [33, 34, 38]. In contrast, assessments of overall sedentary behavior are generally more consistent and uniform [34]. Additionally, many studies rely on categorical measures (e.g., ‘sitting most of the time’ versus ‘hardly ever’), and the absence of more precise, quantified measures may help explain the absence of significant associations between occupational sitting and health outcomes [33].

Despite the inconclusive evidence regarding the impact of occupational sitting on cardiovascular outcomes in the general population, we managed to demonstrate a positive association in our cohort of individuals with T1D. One plausible explanation emerges as we explore the independent mechanisms by which sedentary behavior is hypothesized to impair vascular health. In addition to potential sitting-induced alterations in traditional cardiovascular risk factors (e.g. lipid profile), the hypothesized vascular function impairing mechanisms include metabolic- (e.g., skeletal muscle insulin resistance), inflammatory- (e.g., promotion of low-grade inflammation), and hemodynamic (e.g., reduced shear stress) processes [39, 40]. Simultaneously, individuals with T1D have a pronounced risk of cardiovascular disease due to the inherent nature of the disease, where atherosclerosis occurs at younger age, and the progression is more aggressive [41, 42]. Therefore, one might hypothesize that the cardiovascular risk associated with sedentary behavior may be more pronounced in individuals with T1D, potentially enabling the demonstration of a positive correlation between occupational sitting and cardiovascular events, compared to studies among populations without diabetes. Furthermore, we managed to adjust for key confounders including occupational category, current smoking, and leisure-time physical activity. These adjustments are critical as they have the potential to offset a positive association between occupational sitting and cardiovascular events, as previously discussed and supported by our unadjusted analyses. Finally, using a more quantified measure of sitting time, as recommended by the seminal systematic review on occupational sitting and adverse health outcomes by van Uffelen et al. [33], instead of broad categorical variables like ‘sitting most of the time’ versus ‘hardly ever’, might have helped our study demonstrate a positive association between excessive occupational sitting and cardiovascular events.

In the general population, sedentary behavior is consistently associated with all-cause mortality [6, 7]. This association has recently been replicated in individuals with type 2 diabetes [43]. However, the evidence regarding occupational sitting and all-cause mortality, akin to cardiovascular events, remains inconclusive [33, 34]. While we identified an association between excessive occupational sitting and increased risk of cardiovascular events, we did not observe a corresponding association with all-cause mortality in our cohort of individuals with T1D. There are several plausible explanations for this discrepancy. First, it should be highlighted that our specific focus on occupational sitting led to the exclusion of a significant subset of our initial cohort. Specifically, participants lacking occupational sitting data were excluded, as outlined in the methods section. The predominant reason was individuals on disability pension. Considering the older age and worse health condition among those excluded, especially regarding diabetic complications, our final cohort may have been affected by survival bias. Consequently, this bias might have diminished a potential positive association between excessive occupational sitting and all-cause mortality. If we acknowledge the possibility of a survival bias, it prompts us to question why a positive association between excessive occupational sitting and cardiovascular events persisted. It is conceivable that the deleterious effects of sedentary behavior primarily target the development of cardiovascular events. In essence, although cardiovascular deaths are the leading cause of premature mortality among individuals with T1D [44], the impact of sedentary behavior may have relatively less significance for other causes of premature mortality. Another simplistic rationale is that cardiovascular events typically precede mortality, as not all cardiovascular events result in death, thus mitigating the impact of potential survival bias.

However, among currently smoking individuals with T1D, we managed to demonstrate that excessive occupational sitting increased the risk of all-cause mortality, even after adjusting for leisure-time physical activity, duration of diabetes, occupational category, and diabetic kidney disease. Although a positive trend was observed, the association between occupational sitting and cardiovascular events did not remain statistically significant after final adjustments. In both the general population and individuals with T1D, smoking is associated with cardiovascular events and all-cause mortality [45]. Furthermore, in the general population, smoking has also been shown to exacerbate the harmful effects of sitting [13]. Both behaviors contribute for example to dyslipidemia by inhibiting lipoprotein lipase activity, leading to higher triglyceride levels and lower HDL-cholesterol [39, 46], thus increasing the risk of cardiovascular events and mortality. Consequently, based on biological plausibility and literature, smoking may have accelerated the harmful impacts of occupational sitting in our study cohort, revealing a significant association with all-cause mortality. The lack of statistical significance for cardiovascular events may stem from insufficient statistical power in the analysis, even though a positive trend was observed. In contrast, the significant association with all-cause mortality, observed with comparable statistical power, suggests that smoking may exacerbate the harmful effects of occupational sitting beyond cardiovascular risks, potentially including links to cancer and depression [47–49]. Based on these findings, there might be certain high-risk populations among individuals with T1D, such as smokers, where occupational sitting is particularly hazardous, and targeted interventions needed. In the interpretation of this subanalysis among current smokers, however, it is important to acknowledge the limited number of events.

Finally, in addition to the prospective results, our cross-sectional results are novel, as there is no previous literature on variables independently associated with occupational sitting in individuals with T1D. Perhaps the most intriguing cross-sectional finding in our study was the inverse association between excessive occupational sitting and moderately increased albuminuria. This contrasts with prior, albeit limited, research from the general population reporting a positive association between occupational sitting and proteinuria, and declined kidney health [50, 51]. Notably, we found no significant relationship between more severe kidney conditions and excessive occupational sitting, only with moderate increases in albuminuria.

Several factors may explain our discrepant findings with existing literature. Firstly, our cohort may have experienced survival bias, as a significant number of individuals were excluded due to missing occupational data, particularly those on disability pensions. In contrast, only 9% of participants were excluded for this reason in the study by Tsai et al. [50]. Importantly, our higher exclusion rate reflects reliable measures of participants’ current occupational sitting status, as well as the reduced likelihood of labor force participation among individuals with T1D [52]. Additionally, since other studies were conducted in Asian populations, there could be residual confounding from dietary factors. Diets high in red meat, animal fat, or those with a high acid load—more common in countries like Finland—may adversely affect kidney function, particularly in conditions associated with chronic kidney disease such as T1D [53].

An alternative explanation is that occupational sitting may not significantly influence kidney disease in individuals with T1D. Instead, the observed association between moderate increases in albuminuria and reduced sitting time during work could be attributed transient albuminuria due to various benign causes, including posture, physical activity, and hydration status [54, 55]. For instance, postural albuminuria may be possible in less sedentary, physically active jobs performed in an upright position. Additionally, both strenuous physical activity and dehydration can cause transient albuminuria, both possible in less sedentary jobs. It is unlikely that any single factor can fully explain our observed association. Postural proteinuria, for example, is rare in individuals over 30 years old, at least in the general population [54]. However, individuals with T1D may experience transient proteinuria more easily due to a heightened sensitivity to urinary protein fluctuations. For instance, an exercise-induced increase in urinary albumin excretion rate can occur even with lighter physical activity in individuals with diabetes [55], in contrast to healthy peers, where such effects are typically seen only with more intense physical activity [56].

Major strengths of this study include the nationwide cohort of individuals with T1D and a long follow-up period. Furthermore, the physical activity questionnaire is detailed and has been previously validated in Finnish conditions. We were also able to adjust for important confounders such as occupational category, current smoking, and leisure-time physical activity. In this study, however, several limitations must also be acknowledged. Firstly, the use of a self-report questionnaire introduces the potential for both report and recall bias. While objective tools like accelerometers could mitigate these biases, their use in a large nationwide cohort would have been impractical and poses its own challenges. Another limitation is the inability to examine sedentary behavior patterns in detail. Emerging evidence suggests that not only the total sedentary time but also the pattern of sedentary behavior, such as shorter bouts with frequent breaks, correlates with cardiometabolic markers like postprandial glycemia, insulin responses, and endothelial function [57–59]. In T1D, a recent interventional study demonstrated that interrupting prolonged sitting with light activity every 30 min improved postprandial glucose levels, with benefits lasting up to 48 h [16]. However, while these studies used accelerometers, large-scale studies like ours rely on self-reported data, which significantly limits the ability to reliably capture sedentary patterns, such as the duration of continuous sedentary bouts and the frequency of breaks. Moreover, our study did not account for occupational physical activity (OPA). Additionally, the exclusion of a significant subpopulation from our initial cohort limits the generalizability of our findings to individuals with T1D who are actively employed. Lastly, despite adjustments for important confounders, there remains a risk of residual confounding due to unaccounted lifestyle factors such as diet and alcohol consumption.

## Conclusions

In conclusion, our study finds that excessive occupational sitting is associated with an increased risk of cardiovascular events in individuals with T1D. Additionally, in a subpopulation of current smokers, excessive occupational sitting increased the risk of all-cause mortality. To our knowledge, this is the first prospective study exploring long-term adverse health-outcomes of occupational sitting, or sedentary behavior in general, in individuals with T1D. These findings suggest that current physical activity guidelines need updating to emphasize the reduction of sedentary behavior in individuals with T1D. Given the high prevalence of sedentary behavior among individuals with T1D and the challenges they face in meeting physical activity guidelines—especially in achieving sufficient moderate-to-vigorous physical activity—providing clear recommendations to reduce sedentary behavior could have a significant impact on preventive medicine for this population. Furthermore, occupational sitting, as a specific domain of sedentary behavior, should be considered a key area for intervention. Frequent short breaks, rather than infrequent longer ones, are likely the most health-beneficial way to reduce excessive occupational sitting.

## Electronic supplementary material

Supplementary Material 1

## Data Availability

Individual-level data for the study participants are not publicly available because of the restrictions due to the study consent provided by the participant at the time of data collection. The readers may propose collaboration to research the individual level data with correspondence with the lead investigator.

## Abbreviations

FinnDiane: The Finnish Diabetic Nephropathy Study
LTPA: Leisure-time physical activity
DKD: Diabetic kidney disease
SDR: Severe diabetic retinopathy
