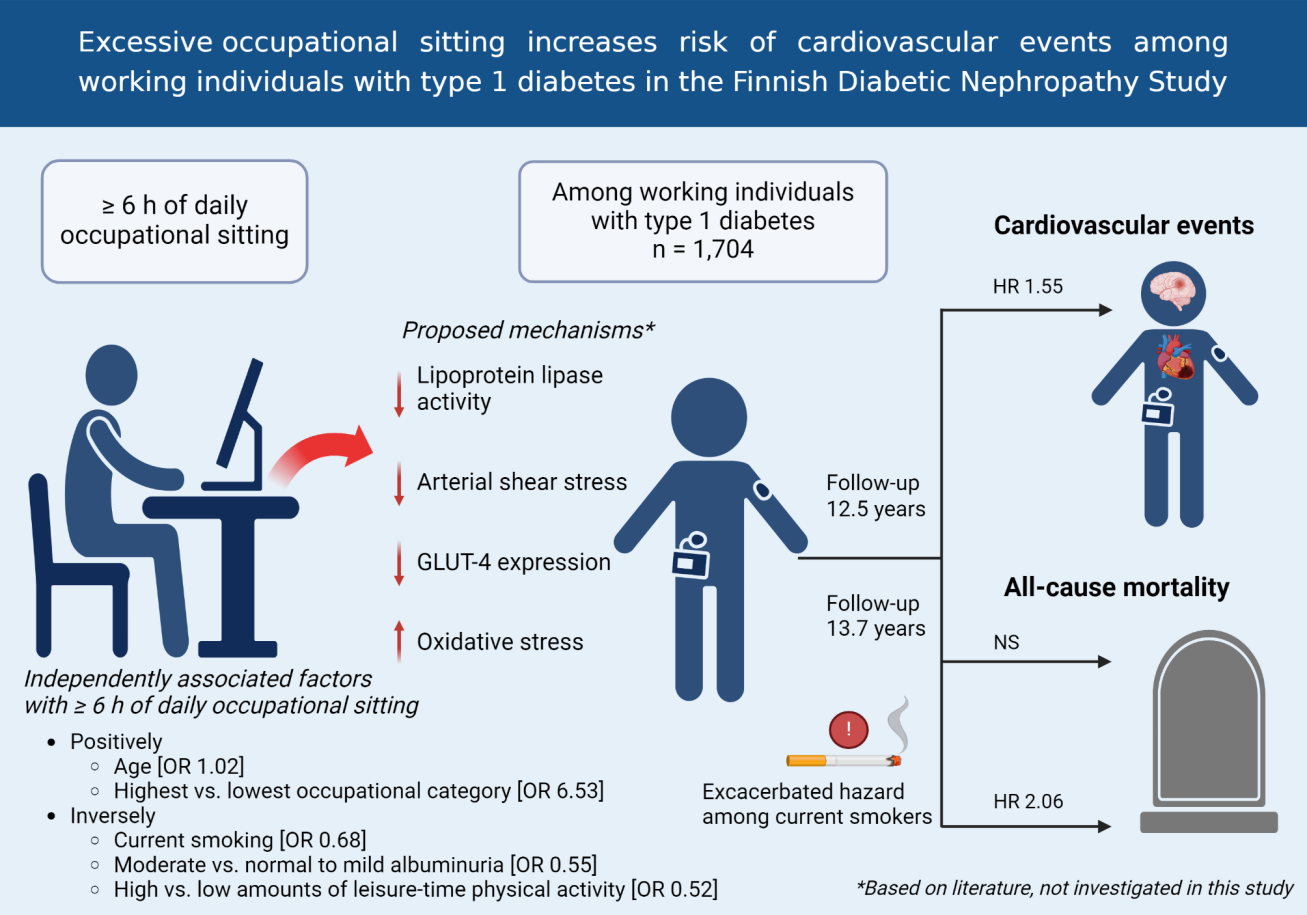# Correction: Excessive occupational sitting increases risk of cardiovascular events among working individuals with type 1 diabetes in the prospective Finnish Diabetic Nephropathy Study

**DOI:** 10.1186/s12933-024-02562-y

**Published:** 2025-01-18

**Authors:** Matias Seppälä, Heidi Lukander, Johan Wadén, Marika I. Eriksson, Valma Harjutsalo, Per-Henrik Groop, Lena M. Thorn, FinnDiane Study Group

**Affiliations:** 1 Folkhälsan Research Center, Biomedicum Helsinki, Helsinki, Finland; 2 Research Program for Clinical and Molecular Metabolism, University of Helsinki, Helsinki, Finland; 3 Department of Nephrology, University of Helsinki and Helsinki University Hospital, Helsinki, Finland; 4 Department of Diabetes, Central Clinical School, Monash University, Melbourne, VIC Australia; 5 Department of General Practice and Primary Health Care, University of Helsinki and Helsinki University Hospital, PoB 20, 00014 Helsinki, Finland

**Correction to: Cardiovascular Diabetology (2024) 23:387** 10.1186/s12933-024-02486-7

Following publication of the original article [1], the graphic abstract was missing from this article and should have appeared as shown below. The original article has been updated.

## Graphical Abstract