# Supplementary material for: Excessive occupational sitting increases risk of cardiovascular events among working individuals with type 1 diabetes in the prospective Finnish Diabetic Nephropathy Study
Source: Cardiovasc Diabetol. 2024 Oct 29;23:387. doi: 10.1186/s12933-024-02486-7 (PMC11520453; doi:10.1186/s12933-024-02486-7)
Supplement: Supplementary file 1 — Supplementary Material 1 [file 12933_2024_2486_MOESM1_ESM.docx]

Supplementary Table 1 – The FinnDiane Study Centers

| Study Center | Physicians and nurses |
| --- | --- |
| Anjalankoski Health Center | S.Koivula, T.Uggeldahl |
| Central Finland Central Hospital, Jyväskylä | T.Forslund, A.Halonen, A.Koistinen, P.Koskiaho, M.Laukkanen, J.Saltevo, M. Tiihonen |
| Central Hospital of Åland Islands, Mariehamn | M. Forsen, H.Granlund, A.-C.Jonsson, B.Nyroos |
| Central Hospital of Kanta-Häme, Hämeenlinna | P.Kinnunen, A.Orvola, T.Salonen, A.Vähänen |
| Central Hospital of Kymenlaakso, Kotka | R.Paldanius, M.Riihelä, L.Ryysy |
| Central Hospital of Länsi-Pohja, Kemi | H.Laukkanen, P.Nyländen, A.Sademies |
| Central Ostrobothnian Hospital District, Kokkola | S.Anderson, B.Asplund, U.Byskata, P.Liedes, M.Kuusela, T.Virkkala |
| City of Espoo Health Center: | |
| Espoonlahti | A.Nikkola, E.Ritola |
| Tapiola | M.Niska, H.Saarinen |
| Samaria | E.Oukko-Ruponen, T.Virtanen |
| Viherlaakso | A.Lyytinen |
| City of Helsinki Health Center: | |
| Puistola | H.Kari, T.Simonen |
| Suutarila | A.Kaprio, J.Kärkkäinen, B.Rantaeskola |
| Töölö | P.Kääriäinen, J.Haaga, A-L.Pietiläinen |
| City of Hyvinkää Health Center | S.Klemetti, T.Nyandoto, E.Rontu, S.Satuli-Autere |
| City of Vantaa Health Center: | |
| Korso | R.Toivonen, H.Virtanen |
| Länsimäki | R.Ahonen, M.Ivaska-Suomela, A.Jauhiainen |
| Martinlaakso | M.Laine, T.Pellonpää, R.Puranen |
| Myyrmäki | A.Airas, J.Laakso, K.Rautavaara |
| Rekola | M.Erola, E.Jatkola |
| Tikkurila | R.Lönnblad, A.Malm, J.Mäkelä, E.Rautamo |
| Heinola Health Center | P.Hentunen, J.Lagerstam |
| Helsinki University Hospital, Department of  Medicine, Division of Nephrology | M.Feodoroff, D.Gordin, O.Heikkilä, T.Claesson, A.Dufva, N.Elonen, M.Eriksson, J.Fagerudd, M.Feodoroff, D.Gordin, O.Heikkilä, K.Hietala, S.Hägg-Holmberg, F.Jansson Sigfrids, M.Korolainen, J.Kytö, S.Lindh, H.Paajanen, K.Pettersson-Fernholm, K.Rimpeläinen, M.Rosengård-Bärlund, M.Rönnback, L.Salovaara, A.Sandelin, M.Saraheimo, S.Satuli-Autere, R.Simonsen, P.Smidtslund, L.Thorn, H.Tikkanen, J.Tuomikangas, A.Tynjälä, K.Uljala, T.Vesisenaho, J.Wadén, A.Ylinen |
| Herttoniemi Hospital, Helsinki | V.Sipilä |
| Hospital of Lounais-Häme, Forssa | T.Kalliomäki, J.Koskelainen, R.Nikkanen, N.Savolainen, H.Sulonen, E.Valtonen |
| Hyvinkää Hospital | L. Norvio, A.Hämäläinen |
| Iisalmi Hospital | E.Toivanen |
| Jokilaakso Hospital, Jämsä | A.Parta, I.Pirttiniemi |
| Jorvi Hospital, Helsinki University Central Hospital | S.Aranko, S.Ervasti, R.Kauppinen-Mäkelin, A.Kuusisto, T.Leppälä, K.Nikkilä, L.Pekkonen |
| Jyväskylä Health Center, Kyllö | K.Nuorva, M.Tiihonen |
| Kainuu Central Hospital, Kajaani | S.Jokelainen, K.Kananen, M.Karjalainen, P.Kemppainen, A-M.Mankinen, A.Reponen, M.Sankari |
| Kerava Health Center | H.Stuckey, P.Suominen |
| Kirkkonummi Health Center | A.Lappalainen, M.Liimatainen, J.Santaholma |
| Kivelä Hospital, Helsinki | A.Aimolahti, E.Huovinen |
| Koskela Hospital, Helsinki | V.Ilkka, M.Lehtimäki |
| Kotka Health Center | E.Pälikkö-Kontinen, A.Vanhanen |
| Kouvola Health Center | E.Koskinen, T.Siitonen |
| Kuopio University Hospital | E.Huttunen, R.Ikäheimo, P.Karhapää, P.Kekäläinen, M.Laakso, T.Lakka, E.Lampainen, L.Moilanen, S. Tanskanen, L.Niskanen, U.Tuovinen, I.Vauhkonen, E.Voutilainen |
| Kuusamo Health Center | T.Kääriäinen, E.Isopoussu |
| Kuusankoski Hospital | E.Kilkki, I.Koskinen, L.Riihelä |
| Laakso Hospital, Helsinki | T.Meriläinen, P.Poukka, R.Savolainen, N.Uhlenius |
| Lahti City Hospital | A.Mäkelä, M.Tanner |
| Lapland Central Hospital, Rovaniemi | L.Hyvärinen, K.Lampela, S.Pöykkö, T.Rompasaari, S.Severinkangas, T.Tulokas |
| Lappeenranta Health Center | P. Erola, L.Härkönen, P.Linkola, T.Pekkanen, I.Pulli, E.Repo |
| Lohja Hospital | T.Granlund, K.Hietanen, M.Porrassalmi, M.Saari, T.Salonen, M.Tiikkainen |
| Länsi-Uusimaa Hospital, Tammisaari | I.-M.Jousmaa, J.Rinne |
| Loimaa Health Center | A.Mäkelä, P.Eloranta |
| Malmi Hospital, Helsinki | H.Lanki, S.Moilanen, M.Tilly-Kiesi |
| Mikkeli Central Hospital | A.Gynther, R.Manninen, P.Nironen, M.Salminen, T.Vänttinen |
| Mänttä Regional Hospital | I.Pirttiniemi, A-M.Hänninen |
| North Karelian Hospital, Joensuu | U-M.Henttula, P.Kekäläinen, M.Pietarinen, A.Rissanen, M.Voutilainen |
| Nurmijärvi Health Center | A.Burgos, K.Urtamo |
| Oulaskangas Hospital, Oulainen | E.Jokelainen, P-L.Jylkkä, E.Kaarlela, J.Vuolaspuro |
| Oulu Health Center | L.Hiltunen, R.Häkkinen, S.Keinänen-Kiukaanniemi |
| Oulu University Hospital | R.Ikäheimo |
| Päijät-Häme Central Hospital | H.Haapamäki, A.Helanterä, S.Hämäläinen, V.Ilvesmäki, H.Miettinen |
| Palokka Health Center | P.Sopanen, L.Welling |
| Pieksämäki Hospital | V.Sevtsenko, M.Tamminen |
| Pietarsaari Hospital | M-L.Holmbäck, B.Isomaa, L.Sarelin |
| Pori City Hospital | P.Ahonen, P.Merisalo, E.Muurinen, K.Sävelä |
| Porvoo Hospital | M.Kallio, B.Rask, S.Rämö |
| Raahe Hospital | A.Holma, M.Honkala, A.Tuomivaara, R.Vainionpää |
| Rauma Hospital | K.Laine, K.Saarinen, T.Salminen |
| Riihimäki Hospital | P.Aalto, E.Immonen, L.Juurinen |
| Salo Hospital | A.Alanko, J.Lapinleimu, P.Rautio, M.Virtanen |
| Satakunta Central Hospital, Pori | M.Asola, M.Juhola, P.Kunelius, M.-L.Lahdenmäki, P.Pääkkönen, M.Rautavirta |
| Savonlinna Central Hospital | T.Pulli, P.Sallinen, M.Taskinen, E.Tolvanen, T.Tuominen, H.Valtonen, A.Vartia, S-L.Viitanen |
| Seinäjoki Central Hospital | O.Antila, E.Korpi-Hyövälti, T.Latvala, E.Leijala, T.Leikkari, M.Punkari N.Rantamäki, H.Vähävuori |
| South Karelia Central Hospital, Lappeenranta | T.Ensala, E.Hussi, R.Härkönen, U.Nyholm, J.Toivanen |
| Tampere Health Center | A.Vaden, P.Alarotu, E.Kujansuu, H.Kirkkopelto-Jokinen, M.Helin, S.Gummerus, L.Calonius, T.Niskanen, T.Kaitala, T.Vatanen |
| Tampere University Hospital | P. Hannula, I.Ala-Houhala, R.Kannisto, T.Kuningas, P.Lampinen, M.Määttä, H.Oksala, T.Oksanen, A.Putila, H.Saha, K.Salonen, H.Tauriainen, S.Tulokas |
| Tiirismaa Health Center, Hollola | T.Kivelä, L.Petlin, L.Savolainen |
| Turku Health Center | A.Artukka, I.Hämäläinen, L.Lehtinen, E.Pyysalo, H.Virtamo, M.Viinikkala, M.Vähätalo |
| Turku University Central Hospital | K.Breitholz, R.Eskola, K.Metsärinne, U.Pietilä, P.Saarinen, R.Tuominen, S.Äyräpää |
| Vaajakoski Health Center | K.Mäkinen, P.Sopanen |
| Valkeakoski Regional Hospital | S.Ojanen, E.Valtonen, H.Ylönen, M.Rautiainen, T.Immonen |
| Vammala Regional Hospital | I.Isomäki, R.Kroneld, L.Mustaniemi, M.Tapiolinna-Mäkelä |
| Vasa Central Hospital | S.Bergkulla, U.Hautamäki, V-A.Myllyniemi, I.Rusk |

Supplementary table 2 – Clinical and socio-demographical characteristics between excluded population and final study population.

| **Characteristic** | **Excluded individuals n = 1,021** | **Final cohort  n = 1,702** | **p-value** |
| --- | --- | --- | --- |
| Men, % (n) | 45.2 (461) | 47.9 (816) | 0.166 |
| Age, years | 42.7 ± 15.2 | 38.9 ± 10.1 | <0.001 |
| Duration of diabetes, years | 27.6 ± 14.3 | 22.3 ± 11.7 | <0.001 |
| BMI, kg/m^2^ | 25.5 ± 3.7 | 25.3 ± 4.2 | 0.152 |
| Systolic blood pressure, mmHg | 138 ± 20 | 133 ± 17 | <0.001 |
| Diastolic blood pressure, mmHg | 78 ± 10 | 79 ± 9 | 0.012 |
| Total cholesterol, mmol/l | 4.77 ± 0.94 | 4.77 ± 0.86 | 0.898 |
| LDL cholesterol, mmol/l | 2.72 ± 0.88 | 2.79 ± 0.79 | 0.056 |
| HDL cholesterol, mmol/l | 1.48 ± 0.42 | 1.48 ± 0.41 | 0.701 |
| Triglycerides, mmol/l | 1.05 (0.79–1.46) | 0.94 (0.73–1.31) | <0.001 |
| HbA1c, % | 8.2 ± 1.4 | 8.1 ± 1.3 | 0.084 |
| HbA1c, mmol/mol | 66.4 ± 15.5 | 65.4 ± 14.3 | 0.085 |
| Retinal photocoagulation, % (n) | 45.4 (461) | 24.6 (417) | <0.001 |
| Diabetic kidney disease, % (n) | 28.3 (264) | 11.6 (197) | <0.001 |
| Cardiovascular event, % (n) | 16.4 (167) | 4.2 (72) | <0.001 |
| Current smoking, % (n) | 17.1 (171) | 22.3 (370) | 0.001 |
| Antihypertensive medication, % (n) | 51.2 (519) | 32.9 (559) | <0.001 |
| Lipid lowering medication, % (n) | 27.7 (280) | 17.0 (288) | <0.001 |
| Kidney status |  |  |  |
| Normal to mildly increased albuminuria, % (n) | 57.9 (540) | 75.6 (1,289) | <0.001 |
| Moderately increased albuminuria, % (n) | 13.8 (129) | 12.8 (218) | 0.453 |
| Severely increased albuminuria, % (n) | 15.2 (142) | 8.8 (150) | <0.001 |
| Kidney failure with replacement therapy, % (n) | 13.1 (122) | 2.8 (47) | <0.001 |
| Educational level |  |  |  |
| Upper white-collar, % (n) | 10.9 (99) | 16.1 (252) | <0.001 |
| Lower white-collar, % (n) | 23.9 (217) | 29.5 (461) | 0.003 |
| Skilled blue-collar, % (n) | 38.4 (349) | 39.5 (615) | 0.639 |
| Unskilled blue-collar, % (n) | 22.0 (200) | 12.5 (196) | <0.001 |
| LTPA |  |  |  |
| High amounts of LTPA, % (n) | 24.5 (250) | 17.8 (303) | <0.001 |
| Moderate amounts of LTPA, % (n) | 49.1 (501) | 53.1 (905) | 0.041 |
| Low amounts of LTPA, % (n) | 26.4 (270) | 29.1 (496) | 0.134 |
| Employment status |  |  |  |
| Unemployed, % (n) | 11.5 (117) | 3.5 (60) | <0.001 |
| Disability pension, % (n) | 31.3 (320) | 0.6 (10) | <0.001 |
| Employment pension, % (n) | 6.6 (67) | 0.1 (1) | <0.001 |
| Rehabilitation subsidy, % (n) | 2.6 (27) | 0.4 (7) | <0.001 |
| Student, % (n) | 10.7 (109) | 2.3 (40) | <0.001 |
| Stay-at-home parent, % (n) | 2.3 (23) | 03 (5) | <0.001 |
|  |  |  |  |

Supplementary Table 3 – Multivariable logistic regression analysis to identify factors independently associated with excessive occupational sitting.

| **Variable** | **Odds ratio** | **95% confidence interval** | **p-value** |  |
| --- | --- | --- | --- | --- |
| **Age** per 1 year | 1.02 | 1.00, 1.03 | 0.009 |  |
| **Skilled blue-collar** vs unskilled blue-collar | 1.43 | 0.93, 2.20 | 0.100 |  |
| **Lower white-collar**  vs unskilled blue-collar | 3.59 | 2.34, 5.52 | <0.001 |  |
| **Upper white-collar** vs unskilled blue-collar | 6.53 | 4.09, 10.40 | <0.001 |  |
| **Current smoking**  yes vs no | | 0.68 | 0.50, 0.92 | 0.012 |
| **Moderate albuminuria**  vs normal to mild albuminuria | 0.55 | 0.38, 0.80 | 0.002 |  |
| **Severe albuminuria** vs normal to mild albuminuria | 1.00 | 0.66, 1.50 | 0.983 |  |
| **Kidney failure with replacement therapy**  vs normal to mild albuminuria | 1.03 | 0.53, 2.00 | 0.935 |  |
| **Moderate amounts of LTPA** vs low amounts of LTPA | 0.91 | 0.70, 1.19 | 0.495 |  |
| **High amounts of LTPA**  vs low amounts of LTPA | 0.52 | 0.36, 0.74 | <0.001 |  |

Data are presented as odds ratio and 95 % confidence interval. LTPA = Leisure-Time Physical Activity.

Supplementary Table 4 – Baseline clinical characteristics according to presence or absence of a given outcome during follow-up.

|  | **Cardiovascular event** | | |  | **All-cause mortality** | | |
| --- | --- | --- | --- | --- | --- | --- | --- |
| **Variable** | **No event** | **Event** | **p-value** |  | **Survivors** | **Non-survivors** | **p-value** |
| Participants, n | 1,453 | 163 | - |  | 1,562 | 108 | - |
| Men, % (n) | 47.0 (683) | 54.6 (89) | 0.066 |  | 47.2 (737) | 63.9 (69) | <0.001 |
| Age, years | 37.8 ± 9.8 | 44.1 ± 8.3 | <0.001 |  | 38.4 ± 9.9 | 46.0 ± 8.9 | <0.001 |
| Duration of diabetes, years | 20.9 ± 11.3 | 29.5 ± 9.1 | <0.001 |  | 21.7 ± 11.5 | 29.5 ± 12.1 | <0.001 |
| BMI, kg/m2 | 25.4 ± 3.6 | 25.7 ± 3.6 | 0.343 |  | 25.5 ± 3.6 | 25.4 ± 4.0 | 0.845 |
| Systolic blood pressure, mmHg | 132 ± 16 | 140 ± 18 | <0.001 |  | 132 ± 16 | 142 ± 20 | <0.001 |
| Diastolic blood pressure mmHg | 79 ± 9 | 80 ± 10 | 0.157 |  | 79 ± 9 | 80 ± 11 | 0.148 |
| Total cholesterol, mmol/l | 4.77 ± 0.83 | 5.01 ± 1.05 | <0.001 |  | 4.76 ± 0.85 | 5.06 ± 0.98 | <0.001 |
| LDL cholesterol, mmol/l | 2.78 ± 0.76 | 3.12 ± 0.91 | <0.001 |  | 2.78 ± 0.77 | 3.01 ± 0.95 | 0.005 |
| HDL cholesterol, mmol/l | 1.50 ± 0.41 | 1.33 ± 0.36 | <0.001 |  | 1.48 ± 0.40 | 1.44 ± 0.45 | 0.391 |
| Triglycerides, mmol/l | 0.93 (0.72–1.29) | 1.09 (0.79–1.52) | <0.001 |  | 0.93 (0.72–1.29) | 1.22 (0.93–1.66) | <0.001 |
| HbA1c, % | 8.1 ± 1.3 | 8.6 ± 1.4 | <0.001 |  | 8.1 ± 1.3 | 8.5 ± 1.5 | 0.001 |
| HbA1c, mmol/mol | 64.8 ± 14.0 | 70.5 ± 15.3 | <0.001 |  | 65.2 ± 14.0 | 69.8 ± 16.1 | 0.001 |
| Retinal photocoagulation, % (n) | 20.7 (298) | 48.1 (78) | <0.001 |  | 22.4 (348) | 53.3 (57) | <0.001 |
| Diabetic kidney disease, % (n) | 8.1 (118) | 30.1 (49) | <0.001 |  | 9.5 (149) | 36.1 (39) | <0.001 |
| Cardiovascular event, % (n) | 0.8 (12) | 4.3 (7) | <0.001 |  | 3.1 (48) | 17.8 (19) | <0.001 |
| Current smoking, % (n) | 22.2 (314) | 27.6 (43) | 0.127 |  | 21.6 (328) | 35.6 (37) | <0.001 |
| Antihypertensive medication, % (n) | 27.7 (401) | 58.9 (96) | <0.001 |  | 30.5 (475) | 63.9 (69) | <0.001 |
| eGFR, ml/min/1.73m^2^ | 106 (93–117) | 95 (70–110) | <0.001 |  | 106 (92–116) | 91 (68–106) | <0.001 |
| Kidney status |  |  |  |  |  |  |  |
| A1, % (n) | 79.8 (1,159) | 48.5 (79) | <0.001 |  | 77.8 (1,216) | 45.4 (49) | <0.001 |
| A2, % (n) | 12.1 (176) | 21.5 (35) | <0.001 |  | 12.6 (197) | 18.5 (20) | 0.077 |
| A3, % (n) | 6.5 (95) | 22.7 (37) | <0.001 |  | 7.6 (119) | 25.9 (28) | <0.001 |
| KFRT, % (n) | 1.6 (23) | 7.4 (12) | <0.001 |  | 1.9 (30) | 10.2 (11) | <0.001 |
| Educational level |  |  |  |  |  |  |  |
| Unskilled blue-collar, % (n) | 10.6 (154) | 20.9 (34) | <0.001 |  | 10.7 (167) | 24.1 (26) | <0.001 |
| Skilled blue-collar, % (n) | 36.3 (527) | 39.9 (65) | 0.365 |  | 36.6 (571) | 37.0 (40) | 0.920 |
| Lower white-collar, % (n) | 27.3 (396) | 22.7 (37) | 0.213 |  | 27.3 (426) | 17.6 (19) | 0.028 |
| Upper white-collar, % (n) | 14.9 (216) | 9.8 (16) | 0.081 |  | 14.7 (230) | 12.0 (13) | 0.444 |
| LTPA |  |  |  |  |  |  |  |
| High amounts of LTPA, % (n) | 18.5 (269) | 12.3 (20) | 0.049 |  | 18.6 (290) | 8.3 (9) | 0.007 |
| Moderate amounts of LTPA, % (n) | 52.8 (767) | 57.1 (93) | 0.300 |  | 53.5 (835) | 46.3 (50) | 0.149 |
| Low amounts of LTPA, % (n) | 28.7 (417) | 30.7 (50) | 0.598 |  | 28.0 (437) | 45.4 (49) | <0.001 |
|  |  |  |  |  |  |  |  |

Data are mean ± standard deviation, median (interquartile range), or percentage (n). eGFR = estimated glomerular filtration rate, A1 = Normal to mildly increased albuminuria, A2 = Moderately increased albuminuria, A3 = Severely increased albuminuria, KFRT = Kidney failure with replacement therapy

Supplementary Figure 1 – Unstratified Kaplan-Meier (A) cardiovascular event-free and (B) survival probabilities for excessive occupational sitting


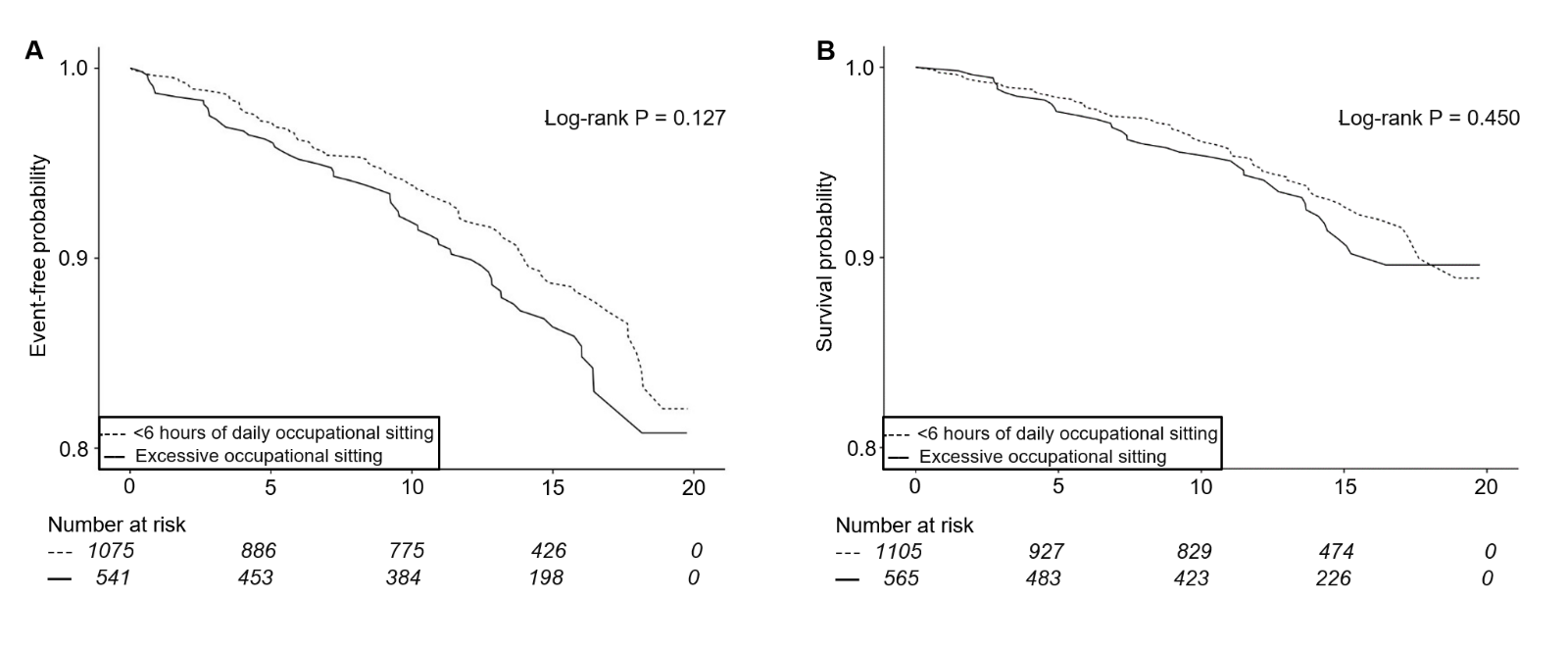


Supplementary Table 5 – Cox regression models showing association between excessive occupational sitting and given outcomes among current smokers.

|  | **Cardiovascular event** | | |  | **All-cause mortality** | | |
| --- | --- | --- | --- | --- | --- | --- | --- |
| **Model** | **n events (n in analysis)** | **HR (95% CI)** | **p-value** |  | **n events  (n in analysis)** | **HR (95% CI)** | **p-value** |
| 1 | 43 (355) | 1.89 (1.01–3.53) | 0.047 |  | 37 (363) | 2.06 (1.06–4.00) | 0.033 |
| 2 | 43 (355) | 1.88 (1.01–3.53) | 0.048 |  | 37 (363) | 2.10 (1.08–4.09) | 0.029 |
| 3 | 43 (355) | 1.93 (1.03–3.62) | 0.041 |  | 37 (363) | 2.16 (1.10–4.21) | 0.025 |

Data are hazard ratios (HR) with 95% confidence intervals (CI).
